# Implementation of fast handover for proxy mobile IPv6: Resolving out-of-order packets

**DOI:** 10.1371/journal.pone.0182375

**Published:** 2017-10-02

**Authors:** Byungseok Kang, Khuong Quoc Anh, Hyunseung Choo

**Affiliations:** 1 Department of Data Science, Sejong Univeristy, Seoul, Korea; 2 College of Software, Sungkyunkwan University, Suwon, Korea; Dalian University of Technology, CHINA

## Abstract

Mobile IP allows for location-independent routing of IP datagrams on the Internet. Mobile IP specifies how a mobile node (MN) registers with its home agent and how the home agent routes datagrams to the MN through the *tunnel*. Current Mobile IP protocols have difficulties meeting the stringent handover delay requirements of future wireless networks. Fast handover for Proxy Mobile IPv6 (FPMIPv6) is used to resolve handover latency and packet loss problems that occur in the Proxy Mobile IPv6 (PMIPv6) protocol. However, while implementing the FPMIPv6 scheme in a testbed, we encounter the out-of-order packet (OoOP) problem. The cause of this problem is the existence of two paths for data transmitted from a correspondent node (CN) to an MN. Since the problem affects the quality of service (QoS) of the network and the performance of the MN, we propose a new scheme using the last packet marker and packet buffering to solve this problem in FPMIPv6. The new Mobile Access Gateway (MAG) can control and deliver the data transmitted via the old path or the new path to an MN in order, using the last packet marker to notify the end of the data delivery in the old path and the packet buffering for holding the data delivered in the new path. We implement both the proposed scheme and FPMIPv6 in a testbed as a real network environment to demonstrate the correctness, cost effectiveness, and performance of the proposed scheme. A performance evaluation reveals that the proposed scheme can handle the OoOP problem efficiently.

## 1. Introduction

IPv6, smartphones, high-speed Internet, and the demand for device-to-device communication everywhere and at any time have all undergone rapid growth. Thus, recent network research has focused on IP mobility management. Mobile IPv6 (MIPv6) [1–2], a host-based mobility management protocol developed by the Internet Engineering Task Force (IETF), has been proposed to enable IP services in the mobile IP network environment. However, the mobile node (MN) of MIPv6 is required to support the mobility protocol stack to handle IP mobility, making the deployment of MIPv6 inefficient in practice.

Proxy Mobile IPv6 (PMIPv6) [3–4] is a network-based mobility management protocol that does not require an MN to be involved in any mobility signaling, making PMIPv6 more practical and efficient. PMIPv6 introduces two entities to handle mobility signaling: a Local Mobility Anchor (LMA) and a Mobile Access Gateway (MAG). LMA maintains the MN’s reachability state and acts as an entry point for the MN’s home network prefix. MAG detects the MN’s movement and registers MN information to the LMA.

Much effort has been expended to bring the mobility management protocol to practice. Universal Mobile IP (UMIP) [5] is an open source implementation of MIPv6 in the Linux environment. It is derived from the GO-Core project [6] in cooperation with the USAGI/WIDE group [7]. Recently, Open Air Interface Proxy Mobile IPv6 (OAI PMIPv6) [8], an open source protocol for the implementation of PMIPv6, has been developed by the EURECOM organization. OAI PMIPv6 is built on top of UMIP in order to reuse the mobility functions.

In this paper, we implement both a fast handover for Proxy Mobile IPv6 (FPMIPv6) and the proposed scheme based on OAI PMIPv6 open source in the Linux environment to measure the performance of the proposed scheme. We set up a testbed with the above scheme to conduct experimental work and evaluate the operation and performance of the proposed scheme. The results from the experiment show that the proposed scheme solves the OoOP problem efficiently and increases the network quality of service (QoS).

In summary, the main contributions of our work are listed as follows:

- Propose a new scheme to solve the OoOP issue in FPMIPv6.
- Implement both FPMIPv6 and the proposed scheme in the Linux environment.
- Develop a testbed to provide IPv6 and wireless communications.

In the following sections, a basic concept of FPMIPv6 is described and implementation information is presented in Section 2. In Section 3, we detail our proposed scheme. Test implementation and experimental results are provided in Sections 4 and 5, respectively. Finally, we conclude the paper in Section 6.

## 2. FPMIPv6 and its implementation

### 2.1 Fast handover for PMIPv6

FPMIPv6 deals with the handover latency and packet loss when an MN roams between MAGs in the PMIPv6 domain by using a proactive approach. [9] FPMIPv6 allows an MN to receive packets from the correspondent node (CN) as soon as the MN enters the new network and the new MAG (nMAG) detects its attachment. According to the Layer 2 report received from the MN, the previous MAG (pMAG) performs the handover initiate (HI) process to establish the tunnel between the pMAG and the nMAG. To prevent a loss of downlink packets for the MN during the handover, the pMAG forwards such packets to the nMAG through the tunnel. The packets are buffered at the nMAG and delivered to the MN as soon as the MN is attached.

The overall signaling process in FPMIPv6 is described as follows. 1) When the RSS of an MN with the pMAG falls below the threshold, the MN scans neighboring MAGs to find the nMAG in which the MN receives a strong RSS. The threshold value is dependent on the MAC layer specification. The MN then sends the Layer 2 report message carrying its MAC address and the nMAG address to the pMAG. 2) Upon receiving the report message, the pMAG starts exchanging HI and Handover Acknowledgement (HAck) messages with the nMAG to establish a tunnel and notice the MN’s information. 3) Once the tunnel is established, the pMAG forwards packets destined for the MN to the nMAG via the tunnel. The nMAG buffers such packets when they arrive. 4) When the MN enters the nMAG’s network, the MN sends a multicast Router Solicitation (RS) message to inform the nMAG of its presence. 5) Once the RS message is received from the MN, the nMAG flushes the buffered packets to the MN. 6) The nMAG exchanges the Proxy Binding Update (PBU) and Proxy Binding Acknowledgement (PBA) messages with the LMA to register the MN’s information and to establish the tunnel between the nMAG and LMA. At the same time, the packets for the MN are delivered directly to the nMAG from the LMA. 7) The nMAG sends an RA message to the MN as a response to the RS message.

When the tunnel between the nMAG and the LMA is established, the route for packet delivery from the CN to the MN is changed at the LMA. At this time, there exist two paths. The first path is an old path that the packets were delivered on via the LMA, the pMAG and the nMAG. The second path is a new path that the packets were delivered on via the LMA and nMAG. The packets destined for the MN are delivered via the two paths at the same time which results in the OoOP problem.

### 2.2. OAI PMIPv6 implementation

OAI PMIPv6 is an open source protocol based on the GNU General Public License version 2 (GPL2). It was developed to bring the PMIPv6 protocol described in RFC 5213 into practice. An OAI PMIPv6 implementation is built on top of UMIP, which is an open source implementation of MIPv6 in the Linux environment. By using this environment, we can easily develop small and large-scale PMIPv6 testbeds. OAI PMIPv6 inherits and reuses the main functions of the mobility of UMIP, such as handling ICMPv6 and mobility messages, as well as tunnel and route management. OAI PMIPv6 has four main modules: Handler, Messages, PMIPv6 Cache, and Finite State Machine (FSM).

The Handler module derives events from Neighbor Discovery Protocol version 6 (NDPv6) [10], and the Mobility Header (MH) and Task Queue modules of UMIP use the interface IHANDLER to handle the incoming ICMPv6 and mobility messages and tasks in the task queue. The Messages module is merely used to generate and send ICMPv6 or mobility messages, and to parse incoming ICMPv6 or mobility messages. The PMIPv6 Cache module is used to maintain the information of MNs in a binding list to manage the mobility of MNs. An FSM is at the heart of OAI PMIPv6. The FSM controls almost all the operations of PMIPv6. It receives events from the Handler module, such as incoming ICMIPv6 or mobility messages, and performs the actions corresponding to such events, e.g., updating the routing table using the Routing Filter module or establishing a tunnel by using the Tunnel Control module of UMIP.

### 2.3. Problem definition

The importance of mobility management is to facilitate the ongoing session and to avoid packet loss in the communication between an MN and a CN when the MN roams into the PMIPv6 domain. FPMIPv6 [11] is proposed to provide continuous service in the PMIPv6 domain. However, FPMIPv6 does not consider the OoOP problem that occurs because FPMIPv6 maintains two paths for data delivery from the CN to MN at the time when the MN has just attached to an nMAG from the pMAG. FPMIPv6 has been extensively researched [12–13], but no one has considered the OoOP problem. Additionally, for the network environment and service provider, the OoOP problem may cause a loss of data in applications using the User Datagram Protocol (UDP) or the increased retransmission of the Transmission Control Protocol (TCP), which reduces the overall network performance or the QoS of the network [14–17].

We propose a new scheme to deal with the OoOP problem in FPMIPv6. In this work, a new mobility message, called the last packet marker (LPM), is proposed to inform the last packet transmitted via the old path. At the time when the LMA starts changing the route for the packets transmitted from the CN to the MN, from the old path to the new path, it sends the LPM message to the nMAG via the old path to notify the end of data delivery via the old path. The packets through the new path are held at the nMAG until the nMAG detects that all the packets through the old path are flushed to the MN by receiving the LPM message. This ensures that all the packets transmitted from the CN are delivered to the MN in the correct order.

## 3. Proposed scheme

In this section, we explain the proposed scheme, in which the nMAG can resolve the OoOP problem based on the awareness of the last packet delivered via the old path. We first introduce the motive for developing the proposed scheme, the LPM method to mark the last packet transmitted via the old path, the signaling procedure, and the format of the LPM message used in the scheme.

### 3.1 Motivation

FPMIPv6 performs a handover of an MN efficiently by reducing the handover latency and minimizing the packet loss. We have implemented FPMIPv6 to bring it to practice. Experimental results in our FPMIPv6 testbed show that the handover latency is remarkably reduced and packet loss is eliminated. However, we reveal that a significant number of packets arrive at the MN out of order.

**Fig 1:**
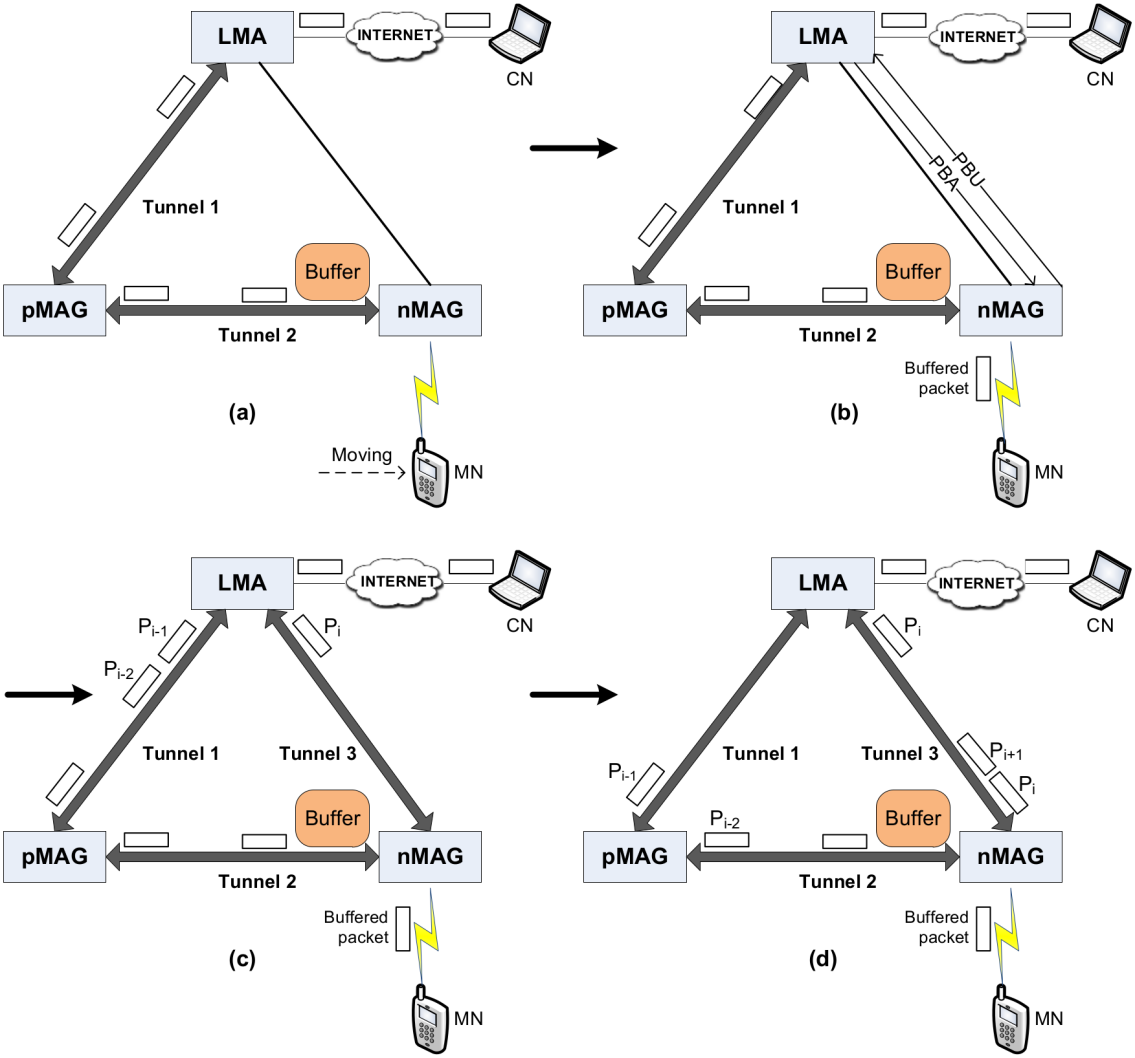
Handover scenario in FPMIPv6.

Fig 1 shows a sequence of images that describes a handover process and expresses the OoOP problem in FPMIPv6. At first, during the movement of the MN from the pMAG to the nMAG, the data packets moving from a CN to the MN are forwarded to and buffered at the nMAG via tunnel 1 and tunnel 2, as shown in Fig 1(a). Once the MN attaches to the nMAG’s network, the nMAG flushes the buffered packets to the MN and then exchanges the PBU and PBA messages with the LMA to establish tunnel 3, as shown in Fig 1(b). When tunnel 3 is ready, the LMA changes the route in its routing table, so that packets from the CN will be delivered to the MN through nMAG using tunnel 3. Suppose that a sequence of packets S = {P_1_, P_2_,…, P_i-1_} is delivered via the old path or tunnels 1 and 2, and a sequence of packets S’ = {P_i_, P_i+1_,…, P_n_} is delivered via the new path or tunnel 3, as shown in Fig 1(c). It is obvious that the first portion of packets {P_i_, P_i+1_,…,P_n_} has higher sequence numbers, but these packets are delivered to the nMAG before the last portion of packets {P_1_, P_2_,…, P_i-1_}, as shown in Fig 1(d). Therefore, the OoOP problem occurs when all the packets arrive at the MN.

The OoOP problem seriously impacts the traffic load in the TCP, due to the retransmission mechanism in the TCP. It also reduces the QoS at the application layer of the MN in the UDP. Therefore, we propose a novel scheme to deal with the OoOP problem in FPMIPv6. The LPM method is used to mark the last of the packets delivered via the old path to notify the nMAG.

### 3.2 Signaling

**Fig 2:**
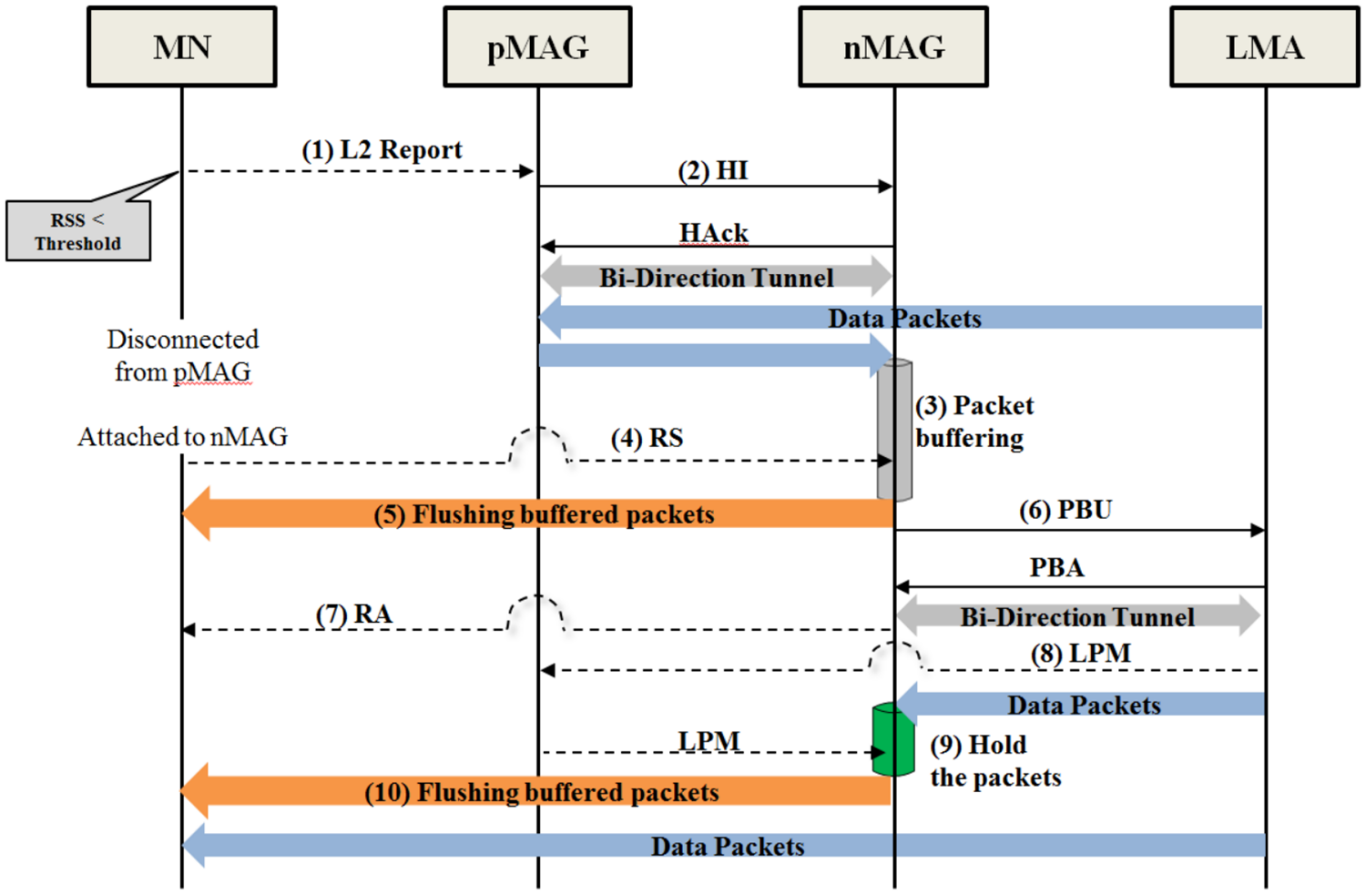
Signaling in the proposed scheme.

We can deal with the OoOP problem in FPMIPv6 with the LPM method. This section describes the signaling procedure in the proposed scheme, as shown in Fig 2, which applies the LPM method to the FPMIPv6 to avoid the OoOP problem.

In the proposed scheme, messages exchanged from steps 1 through 7 are based on FPMIPv6. First, when the RSS of an MN with a pMAG falls below a threshold, the MN scans neighboring MAGs to find the nMAG, and then sends an L2 report message that carries its ID and the address of the nMAG to the pMAG. Upon receiving the report message, the pMAG starts performing the handover initiation procedure by sending the HI and receiving HAck messages to establish a tunnel between the pMAG and the nMAG. When the tunnel is established, the pMAG forwards the data packets to the nMAG through the tunnel. The nMAG buffers the forwarded data packets to prepare for the MN’s attachment. When the MN attaches to the nMAG, it broadcasts an RS message. Upon receiving the RS message, the nMAG flushes the buffered data packets to the MN before it sends the PBU message to register the MN’s information. The nMAG starts to register the MN’s information with the LMA by exchanging PBU and PBA messages with the LMA.

After the tunnel between the LMA and the nMAG is established, the LMA routes packets for the MN to the new path, which delivers the packets via the tunnel between the LMA and the nMAG. At this time, the LMA generates and sends an LPM message to the nMAG via the old path, using the pMAG to mark the last packet via the old path. The nMAG holds the packets transmitted via the new path in the second buffer to avoid the OoOP problem. When the nMAG receives the LPM message, it waits until all packets buffered in the first buffer are flushed. Then, the packets held in the second buffer are flushed to the MN.

According to the proposed scheme, packets for the MN to transmit via the new path are held in the second buffer until two conditions are satisfied. First, the nMAG receives the LPM message to ensure all packets delivered via the old path have arrived. Second, the flushing of buffered packets in the first buffer is complete. Then, the nMAG flushes the packets in the second buffer to the MN, ensuring that the packets arrive at the MN in order. We implement and perform experiments on a testbed to measure the performance and the cost of the proposed scheme. The experimental results show that the proposed scheme can deal with the OoOP problem of FPMIPv6 at a low cost.

### 3.3 The last packet marker

The nMAG has to be aware of the time when the last packet, termed P_i-1_, comes through the old path to prevent OoOPs. To do this, the LMA generates a control message called the last packet marker (LPM), which is a new mobility message, and then sends it to the nMAG via the old path, right after packet P_i-1_ to notify the nMAG that packet P_i-1_ arrived.

**Fig 3:**
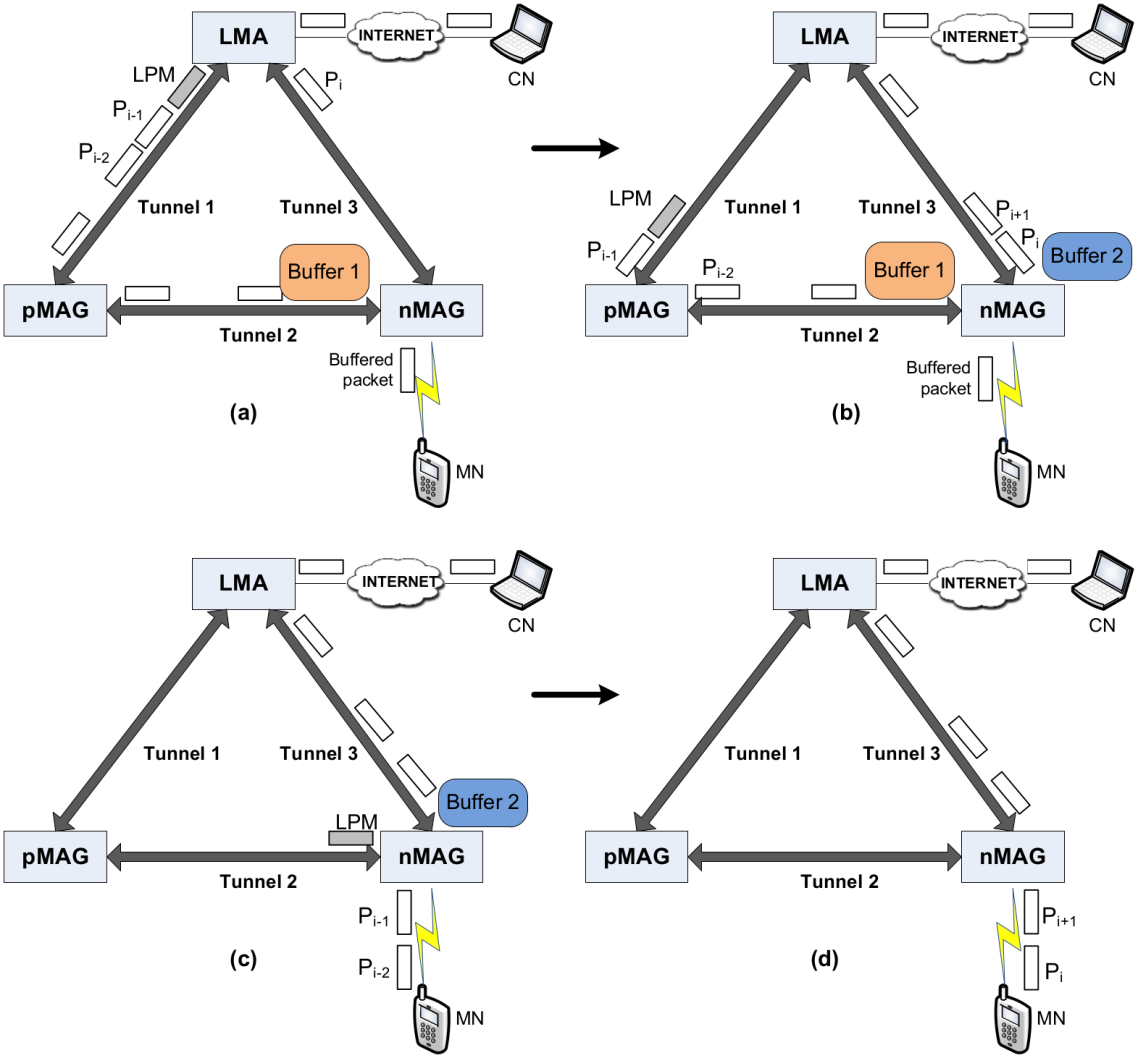
Handover scenario in last packet marker.

Fig 3 shows a sequence of images to describe the operation of the LPM method. As shown in Fig 3(a), the LPM message is transmitted right after the last packet via the old path, P_i-1_. This means that the sequence of packets S = {P_1_, P_2_,…, P_i-1_, LPM} is delivered to the nMAG via the old path, while packet sequence S’ = {P_i_, P_i+1_,…, P_n_} is delivered to the nMAG via the new path. Instead of delivering directly to the MN, a sequence of packets S’ is held in the second buffer to await the LPM message arriving at the nMAG, as shown in Fig 3(b). The packets held in the second buffer are flushed to the MN when the nMAG receives the LPM message and all buffered packets in the first buffer have already been flushed to the MN, as depicted in Fig 3(c). Fig 3(d) depicts the moment packets from the CN are normally transmitted to the MN via the new path.

The LPM method has two main functions. First, generating and sending the LPM message to the nMAG via the old path to mark the end of the sequence packet deliveries. Second, holding the first portion of packet sequence S’ when the packets arrive at the nMAG until the LPM message is received and all buffered packets in the first buffer are flushed completely. These functions guarantee a reliable packet transmission from the CN to MN in sequential order.

### 3.4 Packet format of the last packet marker

The Mobility Extension Header is provided in IPv6 to support the IP mobility protocols. The MH is assigned header code 135, because we need a sequence number ‘out of window’. Currently, 24 MH types from 0 to 23 are defined in the MH [18]. We define a new mobility message, termed the last packet marker (LPM), to mark the last packet that transfers via the old path. The LPM message is assigned header type 24. The LPM message carries the MN address and the nMAG address in the mobility options. The MN address is used to identify the packets transmitted to the MN. The nMAG address is used for the pMAG, which passes the LPM message to the nMAG when it receives it from the LMA.

**Fig 4:**
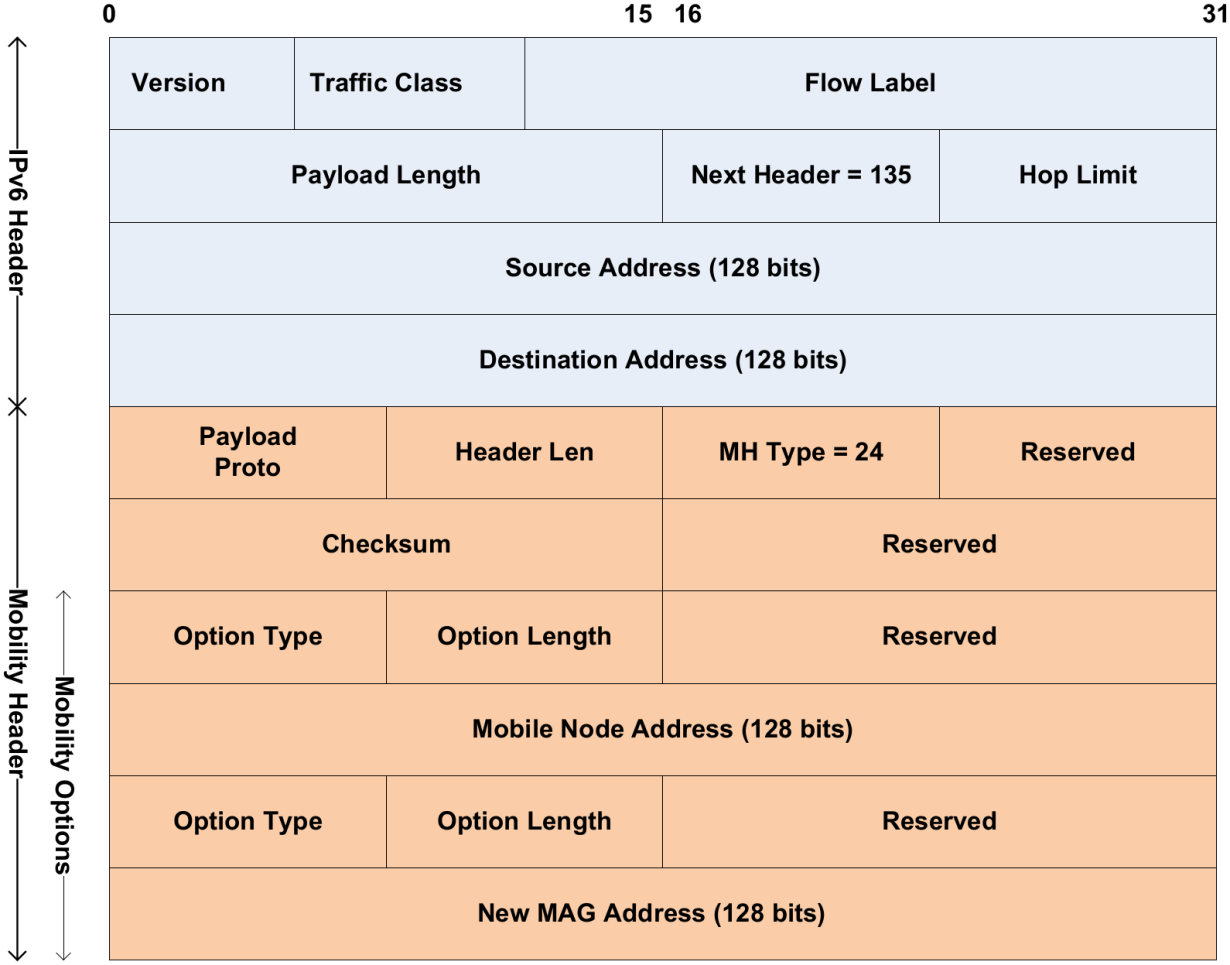
Last packet marker message.

Fig 4 depicts the format of the LPM message that includes the IPv6 header and MH. The next field header in the IPv6 header section is assigned to mobility code 135, which specifies that the next header is an MH. When the LMA generates the LPM message, it uses its address as the source address and the pMAG address as the destination address. When the pMAG receives the LPM message, it clones the message and changes the source address to its address and the destination address to the nMAG address obtained from the mobility options for the LPM message. In the MH, the field MH is assigned type 24 to indicate the LPM message, and the MN address and nMAG address are stored in the mobility options.

### 3.5 Packet buffering

Packets are stored temporarily during the transmission of information to create a reserve for use during packet transmission delays or during a retransmission request. Packet buffering in media systems reduces the effects of packet delays and packet loss. Buffering provides the necessary time to synchronize packets and request and replace those lost during transmission.

**Fig 5:**
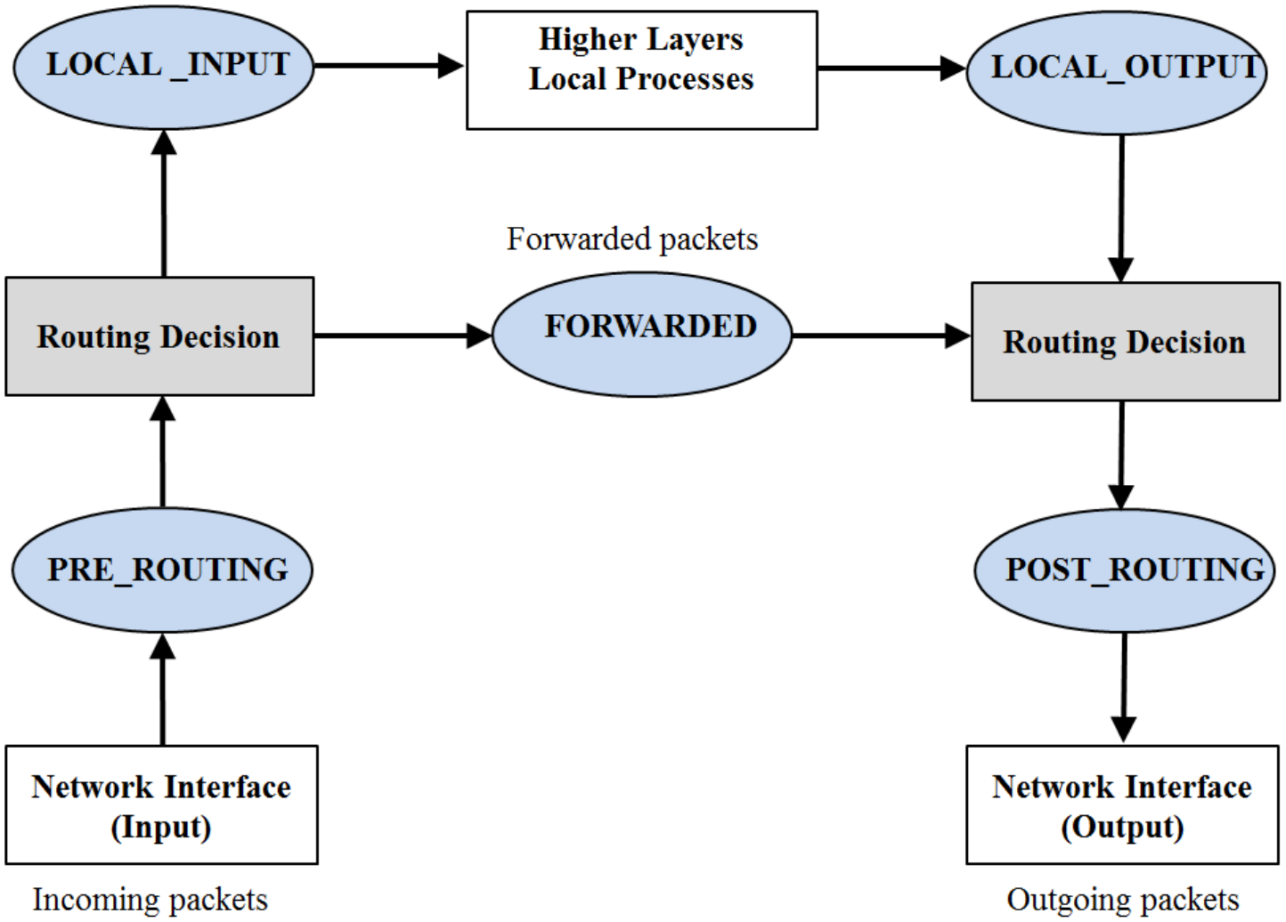
Netfilter hooks and packet flow.

The packet buffering function is used to buffer packets to minimize the number of packets lost during handover of the MN. At the core network in the Linux kernel, the Netfilter framework [19], shown in Fig 5, can be used to intercept packets while they travel in the network stack to implement the packet buffering function [20]. The Netfilter provides hooks, which can be used to intercept packets, on the path of the packets in the network stack. As in Fig 5, Netfilter describes five hooks: PRE_ROUTING, FORWARDED, LOCAL_INPUT, LOCAL_OUTPUT and POST_ROUTING [21]. These hooks stay on different locations on the path of packets in the core network.

First, an incoming packet arrives at the PRE_ROUTING hook from the network interface. The packet then goes to the Routing Decision module that handles the routing of incoming packets. At the Routing Decision module, if the packet is destined for the machine or the local, it is routed to the LOCAL_INPUT hook and then it goes to the higher layers. If the destination of the packet is not the local, the packet is passed to the FORWARDED hook and it goes to the Routing Decision module that describes the route of the outgoing packets. The LOCAL_OUTPUT hook can be used to intercept the outgoing packet from the higher layer before the packet goes to the Routing Decision module. The POST_ROUTING hook is the last hook that an outgoing packet passes through before the packet is transmitted to the network interface to go outside.

The Netfilter framework provides interfaces that allow for implementing the hook handler to intercept packets arriving at the five hooks. A hook handler is a kernel module and it is invoked from the core network code of the Linux kernel. The hook handler can use criteria to filter packets, and the corresponding packets are passed to the handler while the other packets are passed through it. The corresponding packets can be kept and their IP header can be passed up to the application layer by using the IPv6 queue module at the kernel and packet queuing library [22] at the application layer provided by the Netfilter framework.

## 4. Implementation

In this section, we discuss the implementation of FPMIPv6 and the proposed scheme based on the open source OAI PMIPv6. We modify existing modules in OAI PMIPv6 to handle the message processing for FPMIPv6 and the proposed scheme. We also add the new module, Packet Buffering, to buffer the packets in both schemes. We divide the implementation based on the functionalities of the LMA and MAG entities. The implementation is classified into three parts. First, we discuss the structure of the LPM message and the message processing of the LMA. Second, the functions of the pMAG are explained. Finally, we discuss the packet buffering and functions of the nMAG, which are the main implementation of the proposed scheme to avoid the OoOP problem.

### 4.1 LMA functionality

**Fig 6:**
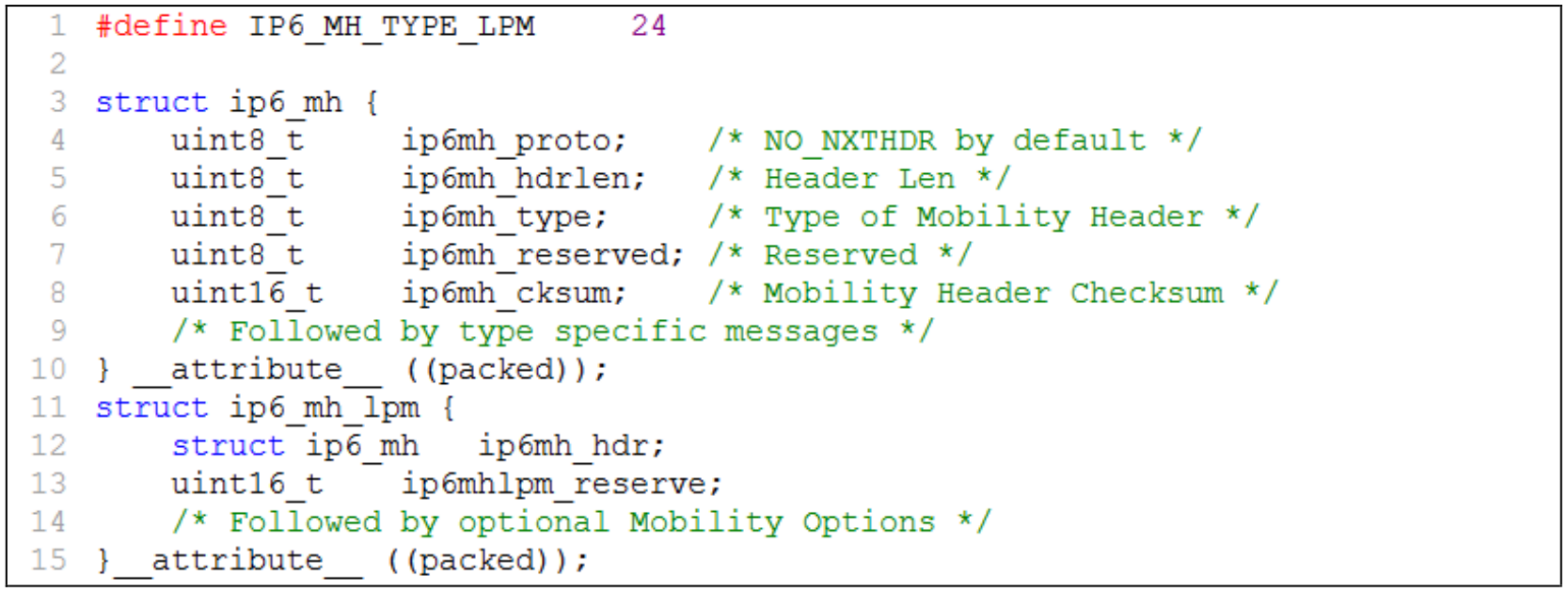
Mobility Header of the LPM message structure.

**Fig 7:**
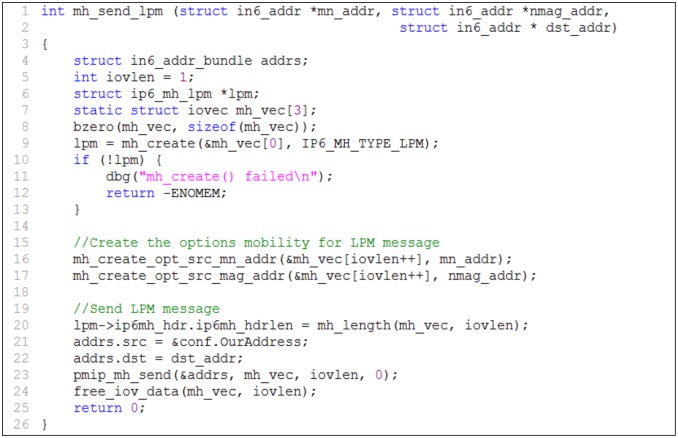
Generating and sending LPM messages.

In the proposed scheme, the main function of the LMA is to generate and send an LPM message when the LMA changes the route of the packet delivery for the MN from the old path to the new path. The MH of the LPM message structure is described in Fig 6. This structure is based on the format of an LPM message. The implementation for generating and sending the LPM message from the LMA to the nMAG via the pMAG is represented in Fig 7.

In the LPM structure, field ip6mh_type represents the MH type (line 6 of Fig 6) of the mobility messages such as PBU, PBA, and LPM. This field is assigned the value 24 to indicate an LPM message. The end of the MH of the LPM message contains the mobility options that store the MN address and the nMAG address. In the implementation, the MH type of an LPM message is created (line 9 of Fig 7) with a value of 24 and the mobility options carry the MN and nMAG addresses (lines 16 and 17 of Fig 7). The message is sent to the pMAG and then forwarded to the nMAG.

The LMA generates and sends an LPM message to the nMAG through the old path when it receives the PBU message from the nMAG to register MN information and establish the new path. Algorithm 1 shows pseudocode for handling a PBU message at the LMA. When the PBU message sent by the nMAG arrives, the LMA checks whether the MN information exists in the binding list or not. If it does exist, that means there will be a handover of the MN from the pMAG to the nMAG.

Algorithm 1. PBU message handling at the LMA.

**INPUT**: *mn_id* is MN ID (MAC address) and *msg_event* is to identify received mobility messages

**OUTPUT**: Handle handover of an MN and send LPM as soon as the LMA changes the route for the MN

1 **If***msg_event* = receive PBU message **then**

2 **   **status ← check *mn_id* exists in binding list

3     **if** status = yes **then**

4  Update the nMAG information instead of the pMAG information in binding entry of the MN

5       Remove the route of MN via the old path

6       Create tunnel LMA-nMAG as a new path

7  Add the route for the MN on which packets are delivered via the new path

8  Generate and send LPM message to the nMAG via the old path

9       Send PBA message to the nMAG

10    **else**

11       Create a new binding entry to register MN information as normal

12    **end if**

13 **end if**

The LMA performs a procedure to handle the handover. The procedure includes updating the binding entry of the MN in the binding list, removing the route via the old path or via the pMAG, creating a tunnel with the nMAG to make a new path, adding the route via the new path, generating and sending an LPM message to the nMAG via the old path to handle the OoOP issue, and finally sending a PBA message as a response to the PBU message to the nMAG (lines 4 through 9 of Algoirthm 1). Based on the algorithm, the LPM message is sent right after the new route is added to switch the delivery of the packet from the old to the new path. This means that the LPM will mark the end of the packet delivery for the MN via the old path.

### 4.2 pMAG functionality

The main function of the pMAG is to exchange HI and HAck messages with the nMAG to establish a tunnel and forward the packets from the MN to the nMAG via the tunnel. In Addition, the pMAG forwards an LPM message to the nMAG when it receives the LMA in the proposed scheme. Algorithm 2 shows the pseudocode for the message handling of the pMAG. The pMAG starts performing a handover by sending an HI message to the nMAG when it receives an L2 report from the MN (lines 1 through 3 of Algorithm 2). The packets for the MN are forwarded to the nMAG when the tunnel between the pMAG and nMAG is established (lines 5 and 6 of Algorithm 2). The last function of the pMAG is to forward the LPM from the LMA to the nMAG to mark the last packet delivered via the old path, LMA-pMAG-nMAG (line 9 of Algorithm 2).

Algorithm 2. Message handling at the pMAG.

**INPUT**: *msg_event* is to identify received messages

**OUTPUT**: Create a tunnel with nMAG and forward LPM message and packets from MN to the nMAG via the tunnel

1 **If***msg_event* = receive L2 report from the MN **then**

2     Get MN information and nMAG address from the L2 report

3     Send HI message to the nMAG to establish a tunnel

4 **else if***msg_event* = receive HAck message from the nMAG **then**

5     Create a tunnel with nMAG

6     Change the route of packet delivery for the MN to the tunnel

7 **else if***msg_event* = receive LPM message from the LMA **then**

8     Get nMAG address from LPM message

9     Forward LPM message to the nMAG

10 **end if**

### 4.3 nMAG functionality

The nMAG plays a key role in FPMIPv6 and the proposed scheme. For FPMIPv6, to avoid packet loss and reduce handover latency, the nMAG buffers the packets forwarded by the pMAG into buffer 1 and flushes such packets as soon as the MN is attached. Furthermore, for the proposed scheme to solve the OoOP problem, the nMAG holds the packet delivered via the new path in buffer 2 until it receives the LPM message and the flushing of all packets in buffer 1 is completed. The implementation is classified into two parts: a packet buffering module and functions of the nMAG.

**Fig 8:**
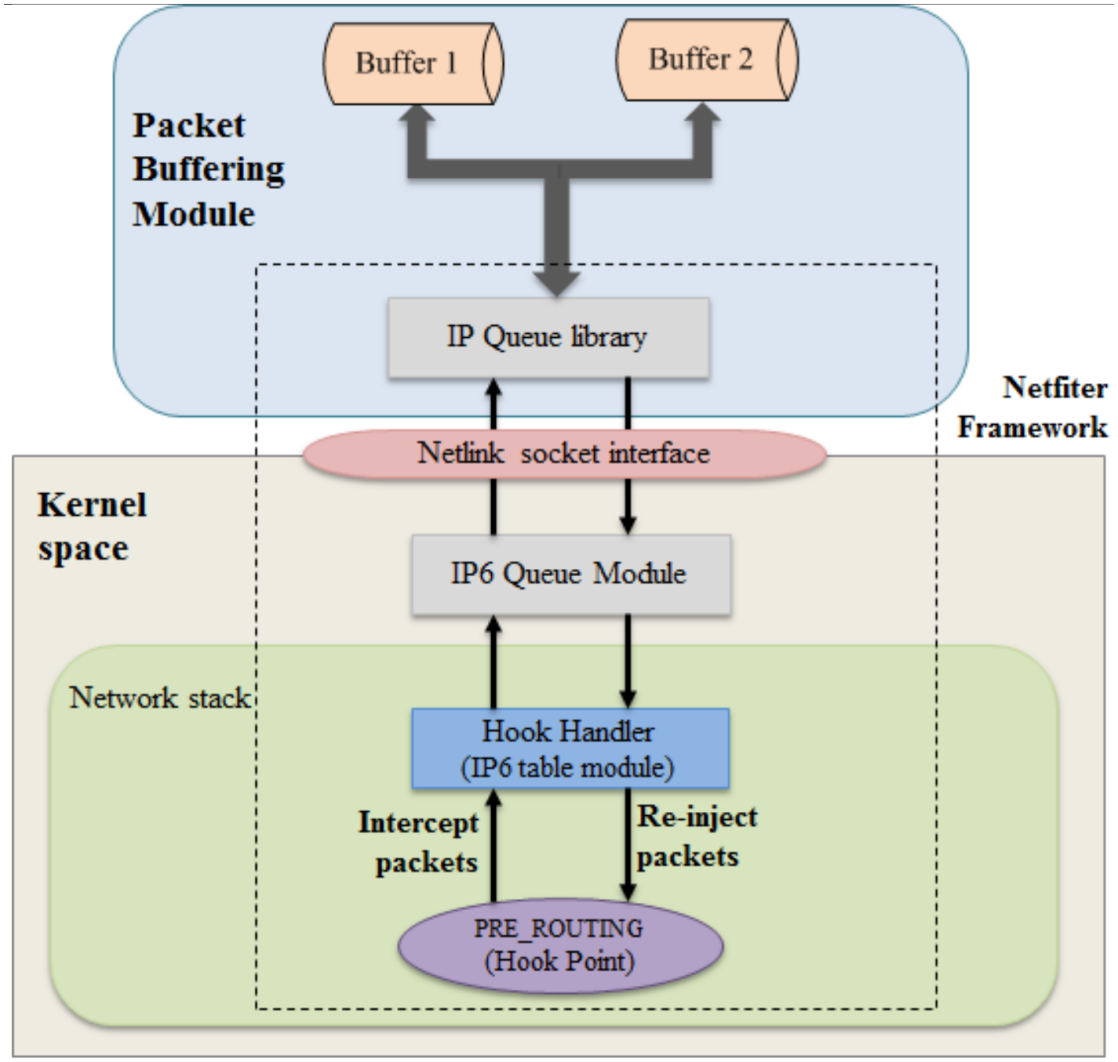
Packet buffering module.

The packet buffering module is used to buffer packets for the MN. The packet buffering implementation based on the Netfilter framework is for intercepting and forwarding the packets from the network stack in the kernel space to the Packet Buffering module in the user space. Fig 8 shows the implementation of packet buffering. The packet buffering implementation intercepts packets at the PRE_ROUTING hook point, which is one of five hooks in the network stack. To hit the packets at the hook point, the implementation uses the IP6 table module provided by the Netfilter framework as a hook handler. The handler filter packets that have the same destination address as the MN address are used to intercept and forward them to the IP6 Queue Module. The packets are stored in a buffer of the IP6 queue Module at the kernel space. The Packet Buffering module uses an IP queue library that remains at the user space to receive and send packets from and to the IP Queue Module at the kernel space. The module stores the packets delivered via the old path or tunnel nMAG-pMAG in buffer 1 and the packets delivered via the new path or tunnel nMAG-LMA in buffer 2.

Algorithm 3 shows the pseudocode to implement the functions of the nMAG. When the nMAG receives the HI message, it creates a tunnel with the pMAG to receive the packets delivered to the MN. The nMAG uses the packet buffering module to intercept and store the packets for MN in buffer 1 (line 3 of Algorithm 3). As soon as the MN attaches to the nMAG, the packets in buffer 1 are flushed to the MN, which reduces the handover latency. The nMAG then sends a PBU message to the LMA to establish a tunnel as a new path for delivering packets to the MN. At the same time, the nMAG creates buffer 2 to buffer the packets for the MN via the new path (line 7 of Algorithm 3). The packets in buffer 2 are flushed to the MN if and only if the nMAG receives the LPM message and the flushing of all packets from buffer 1 to the MN is completed (lines 8 through 14 of Algorithm 3). This ensures that the packets arrive at the MN in order.

Algorithm 3. Message handling at the nMAG.

**INPUT**: *msg_event* is to identify received messages

**OUTPUT**: Avoid packet loss, reduce handover latency and deliver packets to the MN in order

1 **If***msg_event* = receive HI message from the pMAG **then**

2     Create a tunnel with the pMAG

3     Create buffer 1 to buffer packets for the MN forwarded by the pMAG

4 **Else if***msg_event* = attachment of the MN **then**

5     Flush packets from buffer 1 to the MN

6     Send PBU message to the LMA to establish the new path

7     Create buffer 2 to buffer the packets for MN delivered via the new path

8 **else if***msg_event* = receive LPM message **then**

9     **if** buffer 1 is not empty **then**

10         Wait until flushing of packets from buffer 1 is completed

11     **else**

12         Flush packets from buffer 2 to the MN

13     **end if**

14 **end if**

## 5. Experimental results

We perform experiments in a real testbed, comparing the proposed scheme with FPMIPv6 in terms of OoOPs, to evaluate the proposed scheme. The comparison demonstrates that the proposed scheme is able to eliminate the OoOP problem significantly. In addition, we also measure the number of OoOPs in FPMIPv6 and the buffering cost of the proposed scheme.

### 5.1 Experimental environment

**Fig 9:**
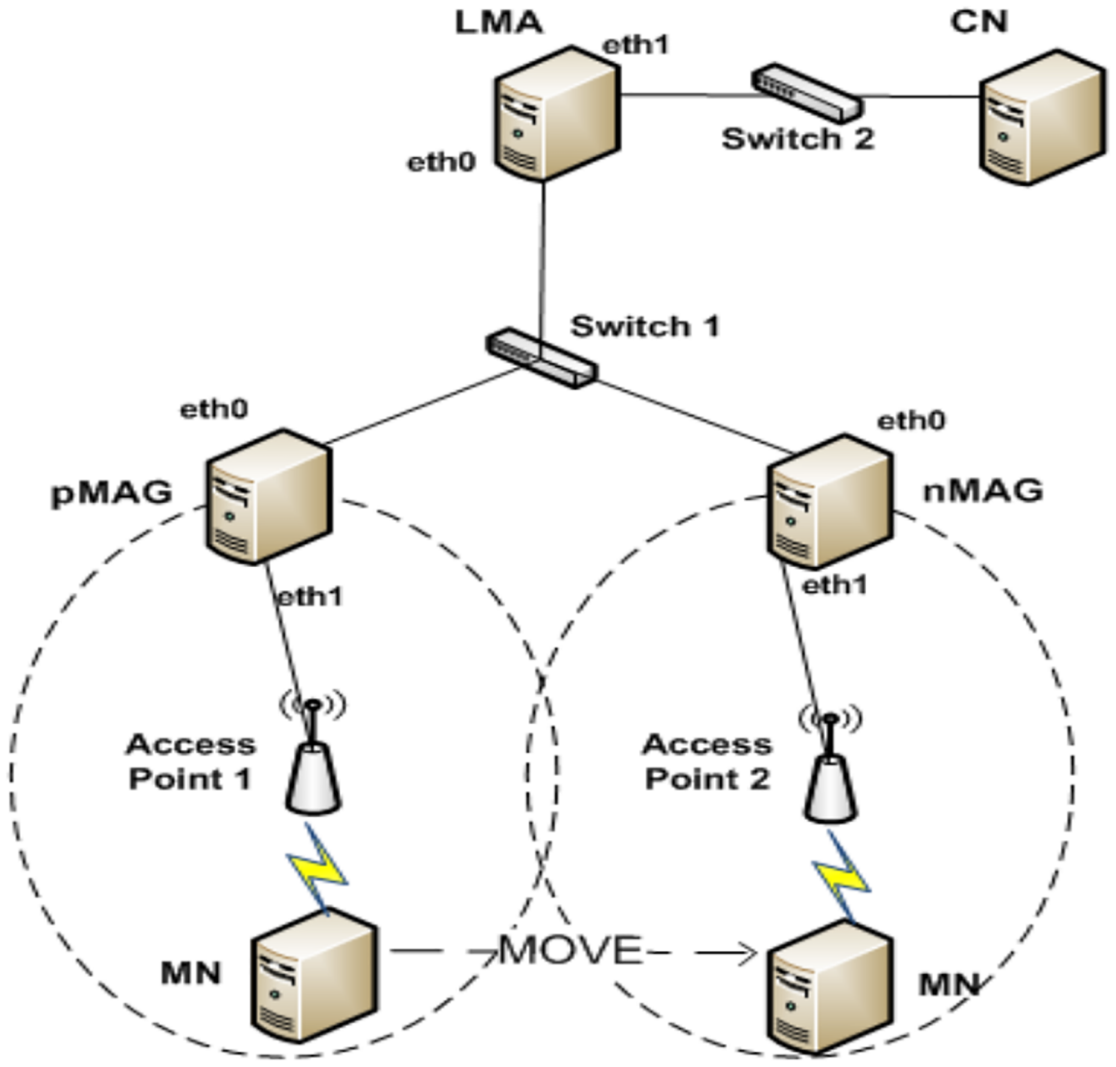
Network topology.

We implement FPMIPv6 and the proposed scheme in Linux based on the open source OAI PMIPv6 to evaluate the performance. Fig 9 describes the network topology used in the experiments. The topology has five mini PCs installed with Linux Ubuntu for the LMA, pMAG, nMAG, MN and CN. All mini PCs are equipped with Intel Core2 Duo 3.0 GHz CPUs and 2 GB of RAM. The Linux kernel in the LMA and two MAGs is recompiled to support the IPv6 mobility function in the network stack. FreeRADIUS software [23] is installed in the LMA for authentication of the MN. We have four IPv6 networks. These are the two home networks of the pMAG and nMAG connected by two access points providing a wireless network for the MN. We have one network to connect the LMA, pMAG and nMAG with switch 1 for the PMIPv6 domain and another to connect the LMA and CN with switch 2.

**Fig 10:**
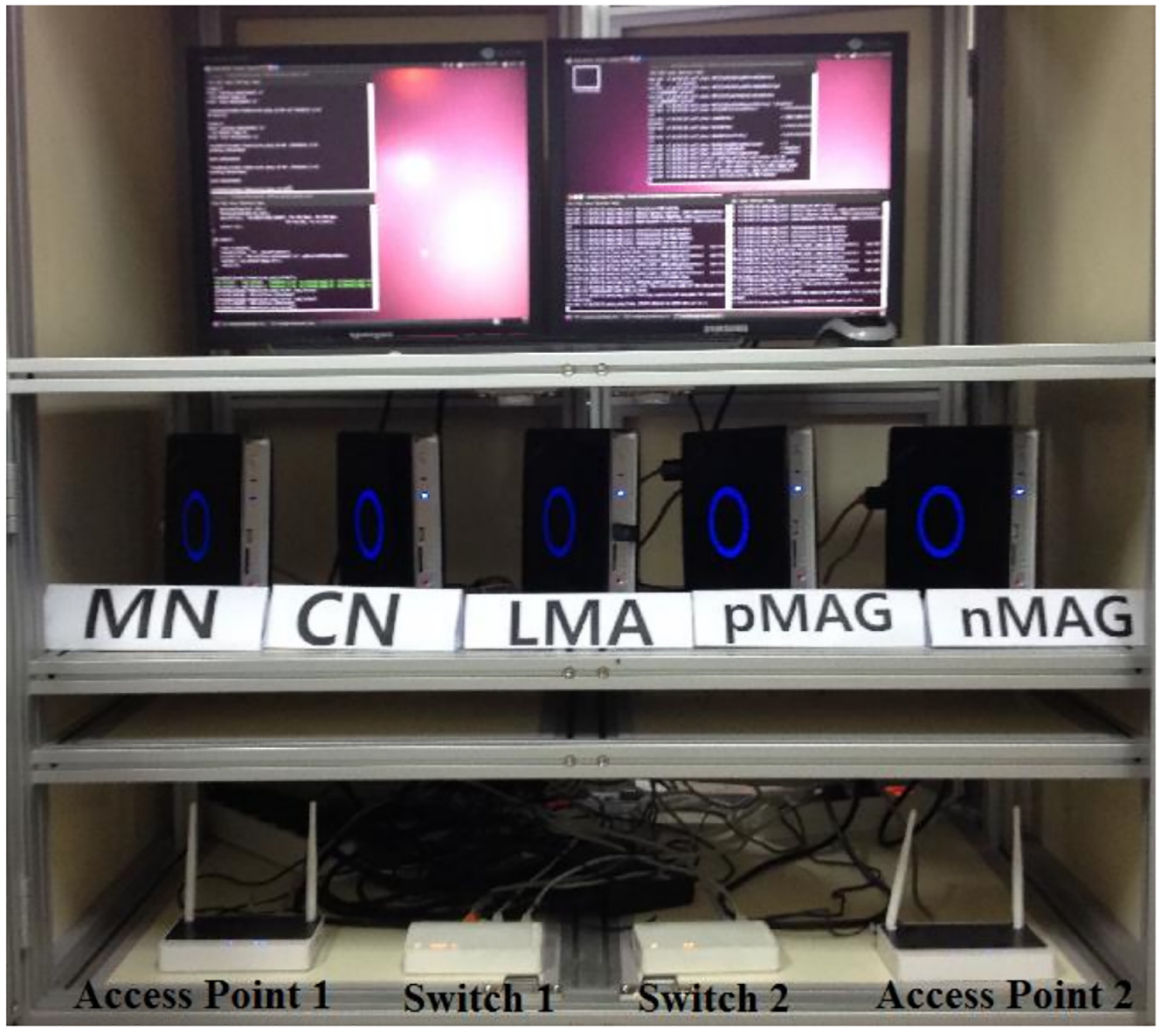
Testbed.

Fig 10 shows the testbed constructed as the network topology in Fig 9. In the experiments, the MN roams from the pMAG to the nMAG and receives packets sent by the CN. We implement a simple UDP client/server application in IPv6 to generate packets from the CN to the MN. The server runs on the CN to send packets to the client running on the MN. Each UDP packet has a fixed size of 1 Kbyte and a sequence number to check the packet order.

### 5.2 Implementation result

**Fig 11:**
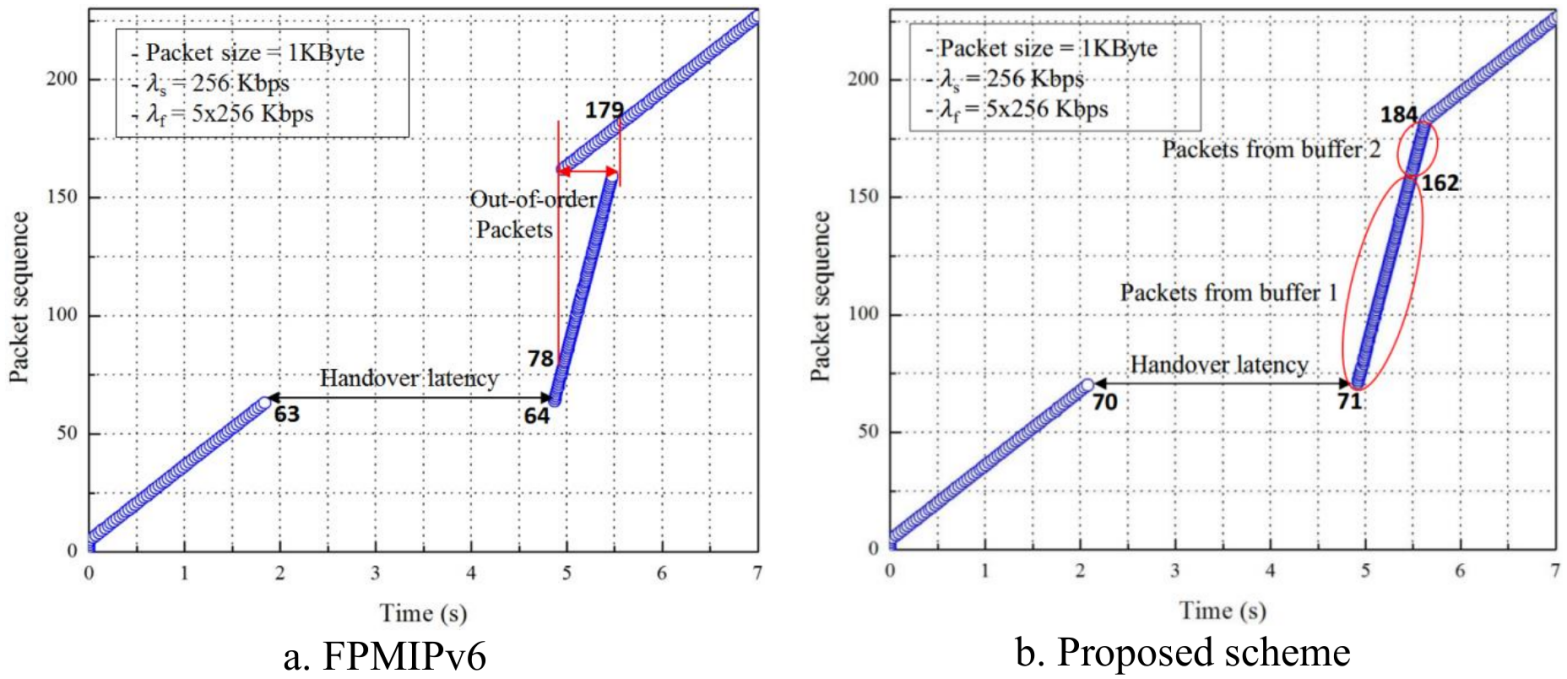
Packet flow with λ_s_ = 256 Kbps, λ_f_ = 5λ_s_. (a) FPMIPv6, (b) Proposed scheme.

Fig 11 shows the sequence of packets at the MN received from the CN in FPMIPv6 and the proposed scheme. The horizontal and vertical axes are time and the packet sequence number that the MN has received, respectively. In this experiment, the sending rate (λ_s_) of the CN is 256 Kbps or 32 Kbps or 32 packets/s. Otherwise, the CN sends packets at 32 packets per second to the MN. The flushing rate of the nMAG (λ_f_) is set to five times λ_s_ or 160 packets/s or the nMAG flushes packets at 160 packets per second from the buffer to the MN. In both schemes, the handover latency is the period between the MN leaving the pMAG and attaching to the nMAG.

Fig 11(a) shows the experiment result of FPMIPv6. The MN receives the 63^rd^ packet before it leaves from the pMAG. After the MN attaches to the nMAG, it receives the 64^th^ packet. Then, we can see that the MN receives the OoOPs in the 78^th^ to 179^th^ packet range. In this range, there exist packets with a higher sequence, but they arrive at the MN before the others, and vice versa. The problem occurs because two delivery paths for packets from the CN to the MN exist, as explained in the proposed scheme section. In contrast, in the experimental result of the proposed scheme, as shown in Fig 11(b), no packets arrive out of order. The MN receives the 71^st^ packet right after attaching to the nMAG. The 162^nd^ to 184^th^ packet range delivered via the new path is held in buffer 2 until the nMAG receives the LPM message and the flushing of packets from buffer 1 is completed. We can see that all packets arrive at the MN in order.

**Fig 12:**
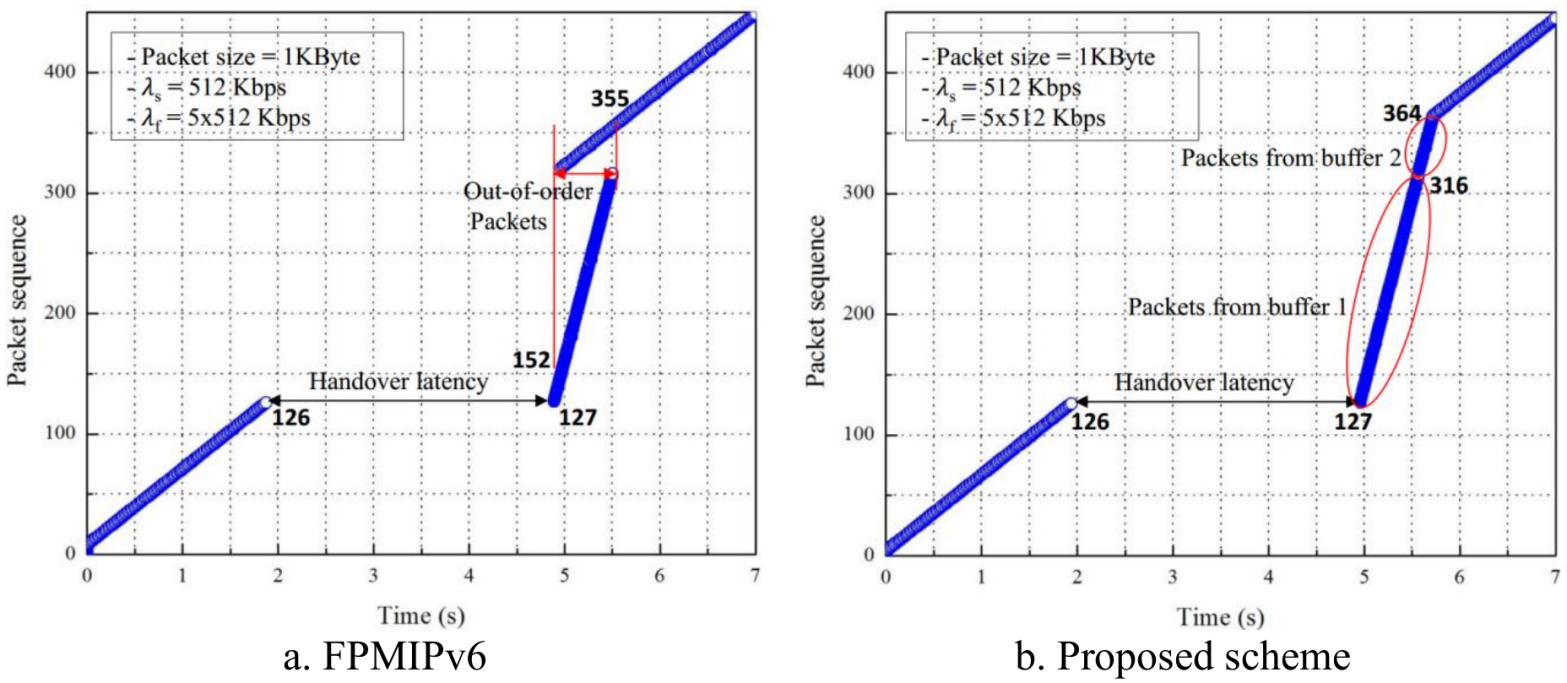
Packet flow with λ_s_ = 512 Kbps, λ_f_ = 5λ_s_. (a) FPMIPv6, (b) Proposed scheme.

We perform the experiment with larger parameter λ_s_. In Fig 12, the CN sends packets at 512 Kbps to the MN and we can see that the density of the packet flow in Fig 12 is higher than it is in Fig 11. Fig 12(a) and 12(b) shows the sequence of packets that arrives at the MN in FPMIPv6 and the proposed scheme, respectively. In Fig 12(a), the OoOP problem occurs in the 152^nd^ and 355^th^ packet range. Compared with the result in Fig 11(a), we can see that the number of OoOPs increases if λ_s_ increases, and vice versa. In contrast, Fig 12(b) shows that the packets arrive at the MN in order in the proposed scheme. The results in Figs 11 and 12 demonstrate that the proposed scheme can efficiently solve the OoOP in FPMIPv6.

**Fig 13:**
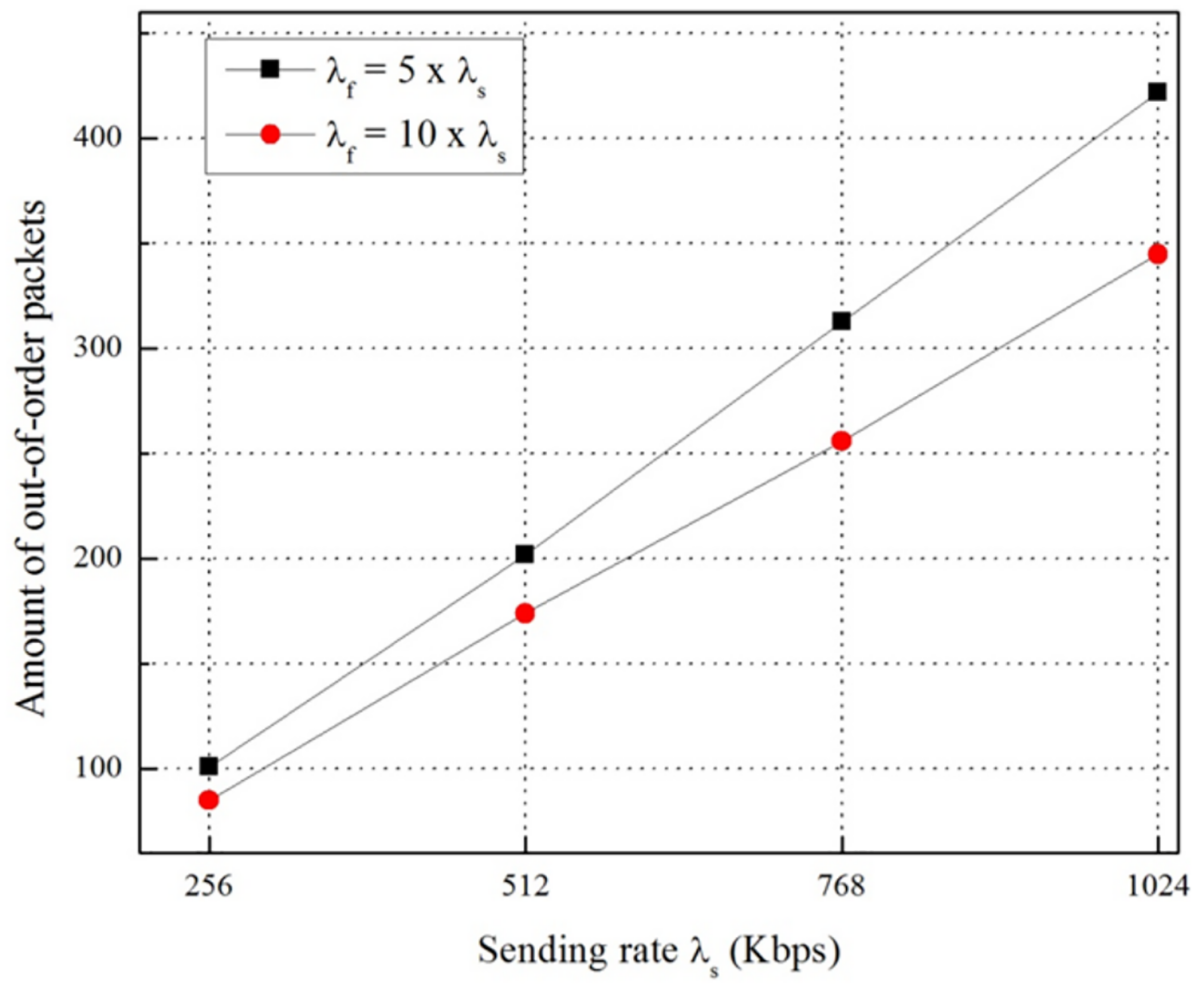
Amount of out-of-order packets in the FPMIPv6 scheme.

Besides experiments regarding OoOPs, we also perform other experiments to evaluate the impact of λ_s_ and λ_f_ on the number of OoOPs in FPMIPv6 and the total number of packets buffered in the proposed scheme for an MN’s handover. Fig 13 shows the number of OoOPs in FPMIPv6 with the parameter sending rate of the CN, λ_s_, and the flushing rate of the nMAG, λ_f_. The result shows that the number of OoOPs increases when λ_s_ increases and λ_f_ decreases and vice versa. That is, it is directly proportional to λ_s_ and inversely proportional to λ_f_.

**Fig 14:**
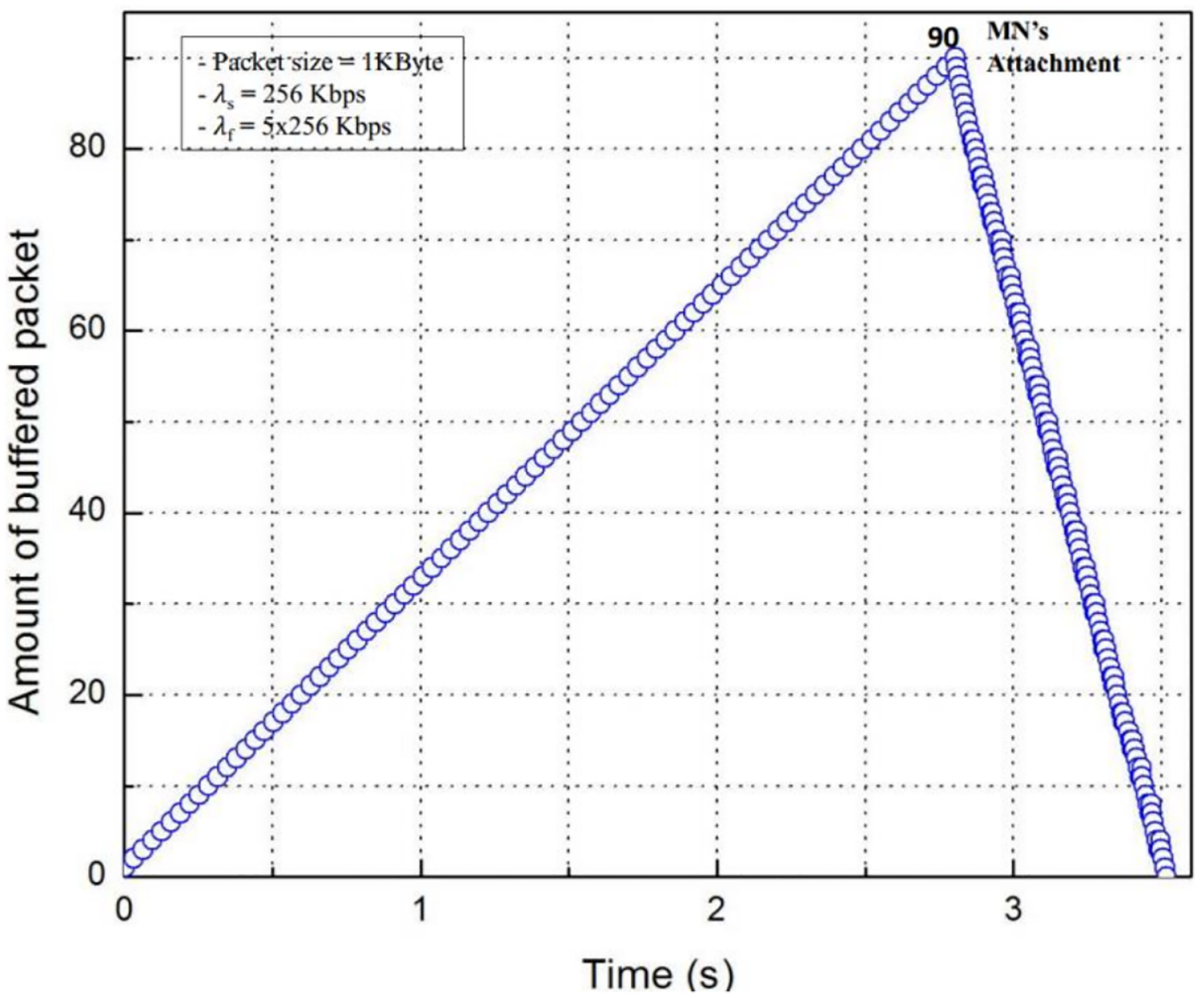
Amount of buffered packets with time variable.

**Fig 15:**
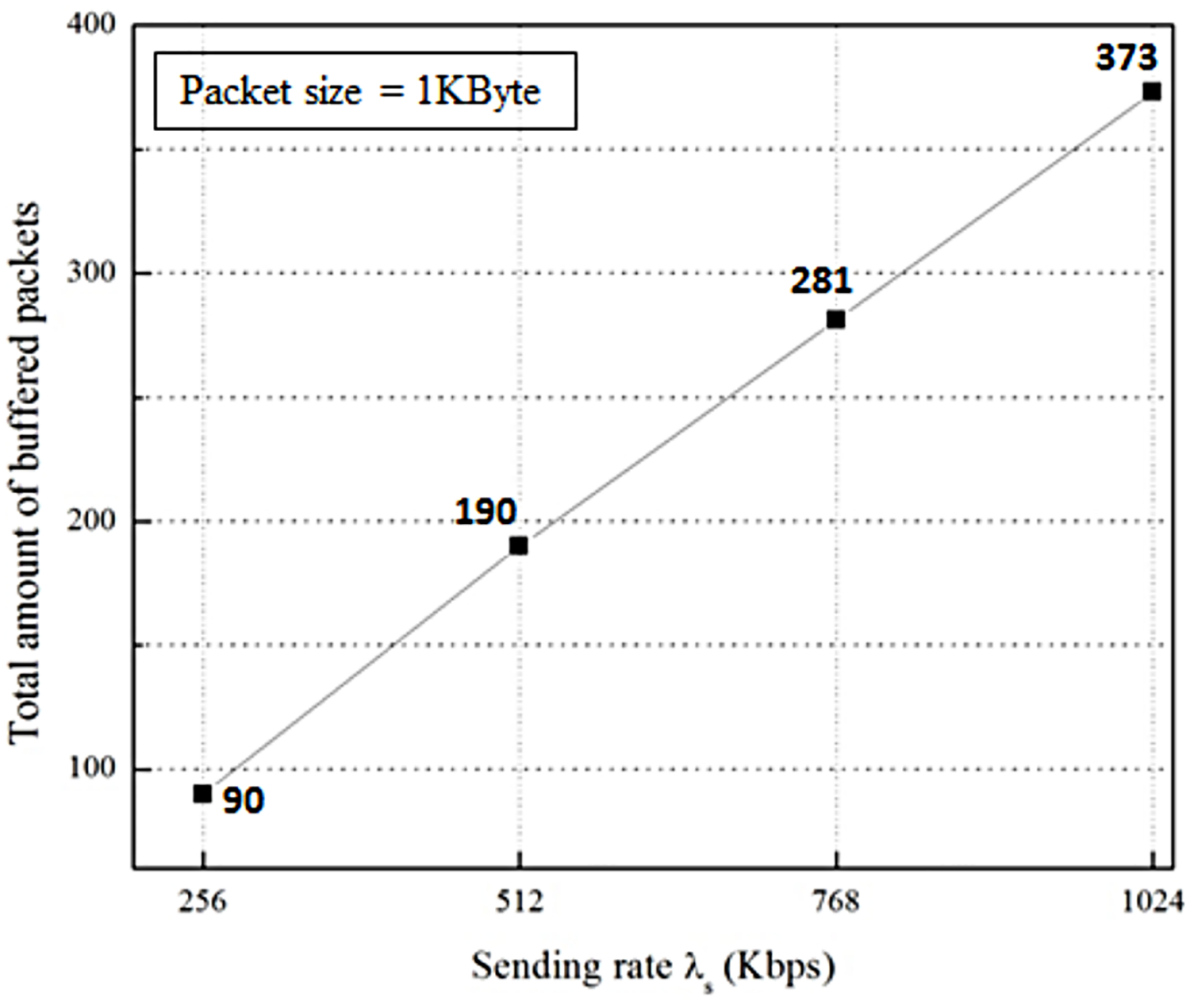
Total amount of buffered packets with variation λ_s_.

Fig 14 shows the buffering cost, which is the number of packets buffered in the proposed scheme for a handover of the MN during the buffering time from the beginning of the packet buffering to the end of the packet flushing, where λ_s_ is 256 Kbps and λ_f_ is 5x256 Kbps. At the time when the MN attaches, the nMAG stores 90 packets, which is the total amount of packets buffered during the buffering time, then the nMAG flushes the buffered packets to the MN. Fig 15 shows the total amount of buffered packets with variant λ_s_. Obviously, this increases when λ_s_ increases. Otherwise, it is directly proportional to λ_s_.

## 6. Conclusion

In this paper, we implemented the FPMIPv6 in the Linux environment based on the OAI PMIPv6 open source protocol. The results of the experiments on the testbed show that the OoOP problem occurs in the handover of the MN in FPMIPv6. Since the problem reduces the QoS of networks and the quality of reliable services, we propose a scheme to solve this problem. The proposed scheme uses an LPM message to notify the last packet delivered through the old path, and a packet buffering function to hold the packets delivered through the new path. We implemented the proposed scheme on our testbed to demonstrate the efficiency and practicality of the proposed scheme in terms of resolving the problem. The experiment results from the testbed prove that the proposed scheme can solve the problem in FPMIPv6 efficiently. Additionally, we measure the impact of variations of the sending rate of the CN, λ_s_, and the flushing rate of the nMAG, λ_f_, on the number of OoOPs in FPMIPv6 and the buffering cost of the proposed scheme. The result shows that the number of OoOPs is directly proportional to λ_s_ and inversely proportional to λ_f_, and the buffering cost is directly proportional to the sending rate, λ_s_.

As ongoing work, we will continue to extend the testbed and implementation. We plan to deploy the testbed in a larger area of the campus using a KOREN network that supports the IPv6 environment. The experiments will be performed with real services, such as voice IP or video streaming, to evaluate the performance of our work [24–25]. Furthermore, we have a plan to compare our scheme with previous protocols in various scenarios.
